# Twenty-Five Years along the Nanometer

**DOI:** 10.1021/acs.nanolett.5c04416

**Published:** 2025-12-04

**Authors:** Joel Henzie

**Affiliations:** † Research Center for Materials Nanoarchitectonics (MANA), National Institute for Materials Science (NIMS), 1-1 Namiki, Tsukuba, Ibaraki 305-0044, Japan

*Nano Letters* became an important place where our shared idiom of scale took shape.

Over 25 years, *Nano Letters* has chronicled the rise of nanoscience. The journal also holds the peculiar distinction of publishing my first paper as a graduate student. Looking back now, I find myself returning to a few early currents that shaped the field, the journal, and also impacted the arc of my career. In 2002, as a graduate student in an organic chemistry program, I stumbled across a viewpoint article by Professor George Whitesides titled “Self-Assembly at All Scales”.^1^ What struck me was not just the topic, but the way the essay transformed “self-assembly” into a shared vocabulary that spanned chemistry, physics, biology, and engineering. It rearranged my thinking: the idea that one principle could provide common ground across such disparate disciplines felt like someone had redrawn the intellectual map I thought I understood. I had also read about George’s “open laboratory” policy, which I took, perhaps naively, as a literal invitation. A few months later, I was in Cambridge, learning from his students and postdocs about soft lithography, microcontact printing, and even the finer points of making espresso. Their quest for simplicity and accessibility in science was compelling: the notion that profound experiments could be carried out with modest, almost improvised tools. That sensibility resonated deeply, and it set me on a different course. Within a few months, I left my organic chemistry program and transferred to Northwestern University in 2003, where I joined the lab of Professor Teri Odom.

I began my research in Teri’s group in late 2003, just a few years after the launch of the U.S. National Nanotechnology Initiative (NNI; January 2000)^2^ and the debut of *Nano Letters* (November 2000).^3^ At the time, “nanoscience” was still a contested label in some corners of academia, wryly dismissed as clever rebranding or “surface science with better tools”. Yet the act of defining a field by a particular length scale—the nanometer—*was* radical in the sense that it provided a unifying language that gave the movement visibility and momentum and, just as importantly, offered a common funding target. Suddenly, chemists, physicists, engineers, and biologists could assemble under the same banner, speaking in a shared idiom of scale—a field evolving under a single unit. *Nano Letters* became an important place where our shared idiom of scale took shape.

My first publication appeared in *Nano Letters* in 2005.^4^ The work seems simple by today’s standards, but we were working in newly cleared ground. If you’ll allow me, I’d like to tell a brief story about how those early experiments shaped my career. In that paper, we described a straightforward way—using photolithography, anisotropic etching, and templated deposition—to fabricate free-standing multimetallic nanopyramids with nanoscale tips (Figure 1A).^5^ The structures were simple to make yet carried incredible power: their anisotropic shape and ultrasharp tips concentrated electromagnetic (EM) fields. An unexpected byproduct of the fabrication process were these large-area (>1 in^2^) free-standing metal films perforated with arrays of nanoholes (Figure 1B). The nanohole arrays became my primary project, which was also published in *Nano Letters*, demonstrating how light coupled with Au and Au/Ni nanohole arrays through surface plasmons (SPs) and showing to what magnitude the SPs mediate enhanced light transmission, at a time when the field of plasmonics was beginning to emerge as a distinct area of research.^6–8^

**Figure 1:**
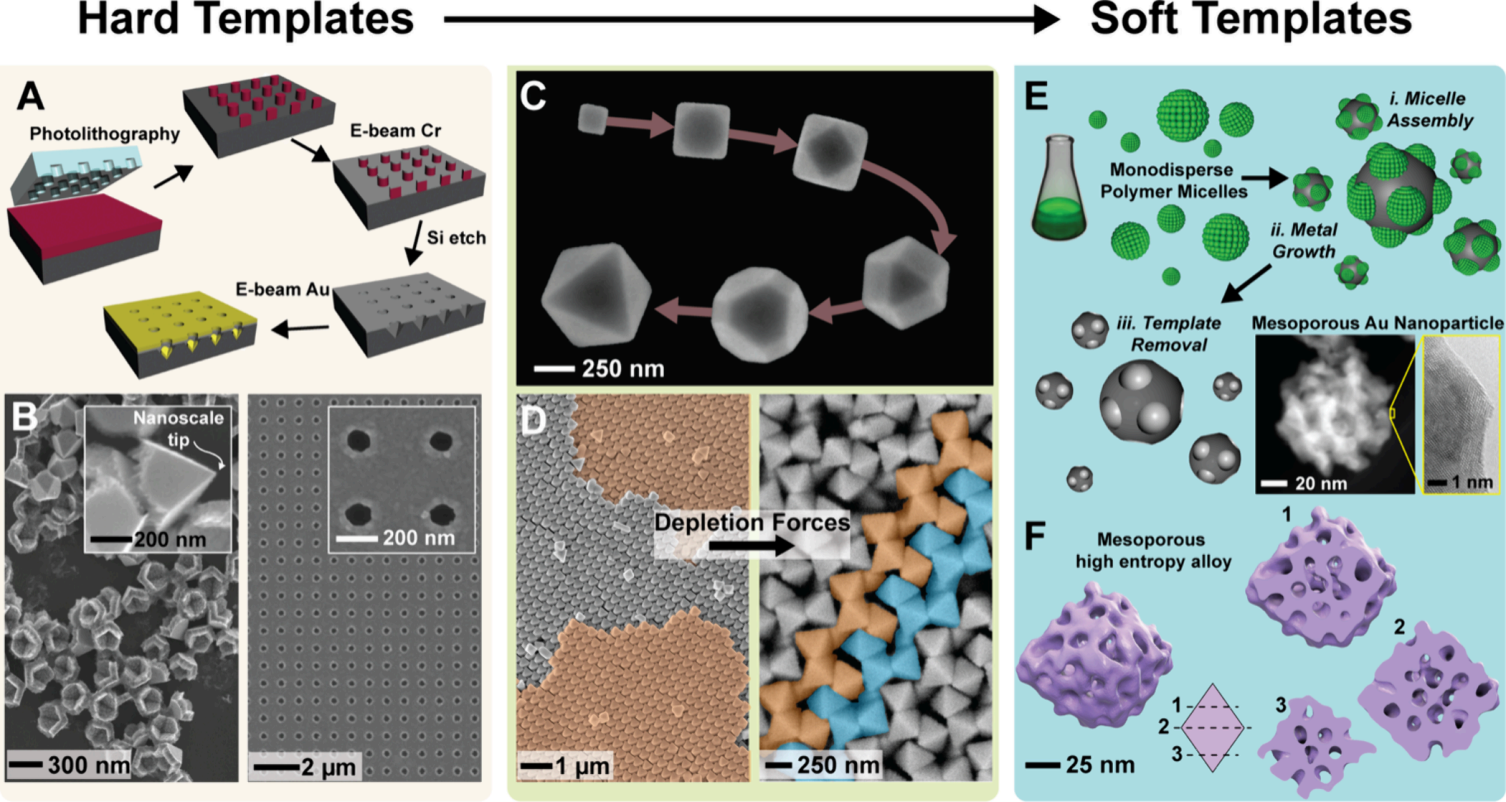
Across the author’s somewhat eclectic journey from plasmonics and nano-optics to electrocatalysis, a unifying theme emerges: the transition from hard to soft-templated metals. (A) Fabrication scheme based on the method described in the author’s first *Nano Letters* paper, resulting in free-standing nanopyramids and nanohole arrays (B). (C) SEM images showing the continuous truncation of nanocubes into intermediate polyhedra and ultimately octahedra via the Ag polyol synthesis method using polyvinylpyrrolidone (PVP) as a surfactant and Ag metal precursor. (D; left) These well-defined shapes were used to explore self-assembly and packing optimization, revealing the densest known arrangement for octahedra—the Minkowski lattice. (D; right) Introducing excess PVP induced depletion forces that favored face-to-face packing, yielding a less dense structure consistent with the *I* 43*d* space group. (E) A schematic of the mesoporous metal synthesis method using block copolymer micelles as pore-directing agents. Removal of the polymer template produces a porous metal framework, illustrated by S/TEM images of a mesoporous gold nanoparticle. This approach is compatible with many metals and even enables the formation of high-entropy alloys, which are metals containing five or more principal elements. (F) A STEM tomography reconstruction of a single-crystal mesoporous PtPdIrRuRh high entropy alloy nanoparticle synthesized using the method in ref 14. The illustration shows the interior pores running through the crystal as it is progressively truncated in the rendered volume (labeled 1 to 3). The schematics and images were reproduced or adapted with permission from ref 4 (Copyright 2005 American Chemical Society), ref 8 (Copyright 2007 Nature Publishing Group), ref 11 (Copyright 2012 Nature Publishing Group), and ref 14 (Copyright 2025 American Chemical Society).

The ideas behind those pyramids and nanoholes—how the structure of metals affects and concentrates EM fields, and how simple fabrication methods can reveal new physics—became a toolkit I carried forward. They also hinted at something I understood but did not yet have the language for: that absence itself could act as an active structure, that the voids shaping EM fields were as important as the metal defining them. As a postdoc in Professor Peidong Yang’s lab at UC Berkeley in 2008, my methods shifted from hard physical templates carved in silicon to soft chemical templates that encode shape on the native crystal habit of the metal. I learned to synthesize monodisperse silver (Ag) nanocrystals with precise polyhedral shapes (Figure 1C), which demanded a deeper understanding of metal redox chemistry and, indirectly, the catalytic properties of metals. We were part of a large, multi-institutional project studying surface-enhanced Raman spectroscopy (SERS), and we used hierarchical self-assembly to pack particles into finite clusters with nanoscale gaps and voids that generated strong EM fields for chemical sensing.^9^ At the time, it seemed natural to assume that larger extended structures would yield even stronger SERS signals, but under the conditions we explored they rarely did. That mismatch between expectation and outcome was characteristic of the era: nanoscience was expanding rapidly, and scientists were trying to pin down the rules linking structure, order, and scale to function. Transient insights were quickly frozen into figures and manuscripts; many of those early attempts found their way into *Nano Letters*, where some have since become part of the field’s foundation.

Around the same time, researchers in statistical physics and materials theory were applying geometric and statistical-mechanical methods to three-dimensional packing problems.^10^ I wondered whether their “optimal packings” were merely abstract constructs or physically accessible states of matter. We used soft lithography to create microfluidic chambers and then relied on gravity to assemble the Ag polyhedra into these densest-known packings (Figure 1D).^11^ With octahedra, adding excess polymer surfactant tipped the balance of forces: depletion interactions encouraged face-to-face packing instead of the edge-overlapping densest Minkowski lattice. Even in these tiny systems, entropy was quietly dictating the architecture of matter. For me, it was a reminder that even modest experiments can reveal how mathematical packings connect to the states matter can actually adopt.

As a student and postdoc in the U.S., I had worked alongside many brilliant foreign researchers, and through them, I caught glimpses of the immigrant experience—with its challenges and dislocation, but also the way it could foster determination and resilience. In 2012, I had the opportunity to experience this firsthand when I moved to Japan to become a staff scientist at the National Institute for Materials Science (NIMS) in Tsukuba. Tsukuba was conceived in the 1960s and 70s as Japan’s ‘Science City’—built from scratch partly to relieve overcrowding in laboratories in Tokyo and to strengthen Japan’s research capacity. Today, Tsukuba hosts 29 national research and educational institutions, plus a dense cluster of industrial R&D facilities. Approximately 20,000 researchers reside and work here, in a city of just a quarter of a million people, where government, academia, and industry are unusually intertwined.

At NIMS, I joined a multidisciplinary team investigating the optical and electrocatalytic properties of metals—an arc of inquiry that ultimately led me to mesoporous metals. My early work on the plasmonics of nanohole arrays and self-assembled nanoparticles had already shown that voids, or “negative space”, were as important as the metal itself. Living and working in Japan, I began to see this principle through the concept of ma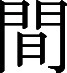, a Japanese aesthetic and philosophical notion of meaningful space, or the interval that gives form and significance to what surrounds it. By using block copolymer micelles, our group learned to control voids in 3D: either as electrodeposited films on complex metal templates for SERS sensing of microplastics,^12^ or as internal cavities within nanoparticles to tune their plasmon modes (Figure 1E).^13^ What began as a byproduct of making pyramids evolved into a guiding design principle: space is not absence but a functional element. Our group extended this approach across different metals and, most recently, demonstrated mesoporous single-crystal high-entropy alloys with remarkable electrocatalytic activity (Figure 1F).^14^ Looking back, I see these advances as natural descendants of early work published in *Nano Letters*, which helped highlight how space and structure could be explored at the nanoscale.

It has been two decades since my first publication in *Nano Letters*. In that time, I developed a deeper appreciation for organic chemistry. And nanoscience tools have become embedded in medicine, energy, information technology, and security. With that success has come new responsibilities and oversight of ideas, of knowledge, of talent moving across the globe. Still, in this new environment, it is worth remembering how much of our early progress stemmed from a spirit of openness that attracted talent from across the world, and the field grew richer by integrating perspectives that no single discipline or country could have provided alone. That spirit is still visible in the pages of *Nano Letters*, and preserving it may be the key to the field’s next transformation.
